# Affective states and stress in health-professional caregivers of people with functional diversity: the important role of empathy and social support as mediators of this relationship

**DOI:** 10.17533/udea.iee.v43n1e12

**Published:** 2025-04-29

**Authors:** Elvira García-Marín, Marián Pérez-Marín, Ana Martínez-Cuevas, Selene Valero-Moreno

**Affiliations:** 1 M.Sc. Student and Psychologist. Email: elgarma2@alumni.uv.es https://orcid.org/0009-0007-7960-5836 Universitat de Valencia Spain elgarma2@alumni.uv.es; 2 Associate Professor. Email: marian.perez@uv.es https://orcid.org/0000-0003-3532-8818 Universitat de Valencia Spain marian.perez@uv.es; 3 Social Worker. Email: amcuevas2@hotmail.com https://orcid.org/0000-0002-8674-6756 Institut Valencià de Serveis Socials Spain amcuevas2@hotmail.com; 5 University of Valencia, Faculty of Psychology and Speech Therapy, Personality, Assessment and Psychological Treatment Department. Valencia (Spain). https://orcid.org/0000-0002-5228-2738 Universitat de Valencia University of Valencia Faculty of Psychology and Speech Therapy, Personality, Assessment and Psychological Treatment Department Valencia Spain; 6 Institut Valencià de Serveis Socials (IVASS), Spain. Institut Valencià de Serveis Socials Institut Valencià de Serveis Socials (IVASS) Spain

**Keywords:** caregiver burden, intellectual disability, stress, empathy, social support, emotional regulation., carga del cuidador, discapacidad intelectual, stress, empatia, suporte social, regulación emocional., carga do prestador de cuidados, deficiência intelectual, stress, empatia, apoio social, regulação emocional.

## Abstract

**Objective.:**

The aim of this study was to analyse how empathy and social support mediate levels of perceived and relational stress, depending on the person's type of affect.

**Methods.:**

This was a multicentre, cross-sectional, descriptive study. The sample consisted of 756 working health professionals for people with functional diversity in the province of Valencia. The following scales were analysed: (i) Perceived Stress Scale; (ii) The Nurse Stress Scale; (iii): Empathy Quotient (EQ); (iv) Social Support Questionnaire, and (v) Scale of Positive and Negative Affects. Descriptive statistics, reliability tests, comparison of means, correlation coefficients and PROCESS were performed.

**Results.:**

The findings showed that stress was positively correlated with negative affects, and negatively correlated with empathy, social support and positive affects, and that these variables influence perceived stress, significantly reducing its levels, while the effect of these variables on relational stress was not significant.

**Conclusion.:**

The data obtained highlights the importance of empathy and a good social support network in these professionals and how this will influence the care and relationship with the users.

## Resumen

**Objetivo.:**

El objetivo de este estudio fue analizar cómo la empatía y el apoyo social median en los niveles de estrés percibido y relacional, dependiendo del tipo de afecto de la persona. Método. Se trata de un estudio multicéntrico, transversal y descriptivo.

**Métodos.:**

La muestra fue de 756 profesionales sanitarios que atienden a personas con diversidad funcional de la provincia de Valencia. Se analizaron las siguientes escalas: (i) Escala de estrés percibido; (ii) Escala de estrés relacional en enfermería (iii): Cociente de Empatía (EQ); (iv) Cuestionario de apoyo social, y (v) Escala de afectos positivos y negativos. Se realizaron estadísticos descriptivos, pruebas de fiabilidad, comparación de medias, coeficientes de correlación y PROCESS**.**

**Resultados.:**

Los resultados mostraron que el estrés se correlacionó positivamente con los afectos negativos, y negativamente con la empatía, el apoyo social y los afectos positivos, y que estas variables influyen en el estrés percibido, reduciendo significativamente sus niveles, mientras que el efecto de estas variables sobre el estrés relacional no fue significativo.

**Conclusiones.:**

Los datos obtenidos ponen de manifiesto la importancia de mostrar empatía y una buena red de apoyo social en estos profesionales y cómo esto influirá en la atención y relación con los usuarios.

## Resumo

**Objetivo.:**

O objetivo deste estudo foi analisar como a empatia e o apoio social mediam os níveis de estresse percebido e relacional, dependendo do tipo de afeto da pessoa.

**Método.:**

Trata-se de um estudo multicêntrico, transversal e descritivo. Métodos. A amostra foi composta por 756 profissionais de saúde que atendem pessoas com diversidade funcional na província de Valência. Foram analisadas as seguintes escalas: (i) Escala de estresse percebido; (ii) Escala de estresse de enfermagem; (iii): Quociente de Empatia (QE); (iv) Questionário de apoio social e (v) Escala de experiências positivas e negativas. Foram realizadas estatísticas descritivas, testes de confiabilidade, comparação de médias, coeficientes de correlação e PROCESS.

**Resultados.:**

Os resultados mostraram que o estresse foi positivamente correlacionado com o afeto negativo e negativamente correlacionado com a empatia, o apoio social e o afeto positivo, e que essas variáveis ​​influenciaram o estresse percebido, reduzindo significativamente seus níveis, enquanto o efeito dessas variáveis ​​no estresse relacional não foi significativo.

**Conclusões.:**

Os dados obtidos mostram a importância de demonstrar empatia e uma boa rede de apoio social nesses profissionais e como isso influenciará no cuidado e no relacionamento com os usuários

## Introduction

Long-term care tasks in healthcare services are primarily performed by professional caregivers. Professional caregivers are individuals external to the family nucleus who possess training in caregiving skills and receive financial remuneration for their work health professionals, including nurses, psychologists, assistants, orderlies, occupational therapists, social workers.^1^This professional tasks entails performing tasks that address the demands of users and services, typically resulting in high levels of stress that negatively impact both the caregiver and the person receiving care, hindering the development of healthy caregiving relationships.^2^The various tasks involved in the professional care of people with functional diversity often lead to significant psychological distress, such as loss of interest, low self-esteem, feelings of guilt, fatigue, or lack of concentration, among other symptoms.^3^Consequently, these professionals often exhibit significant psychological sequelae that affect their ability to cope healthily with their daily work, with stress being a central clinical element inherent in their experience of work life.

In the care of people with functional diversity, these stress levels vary mainly depending on the type of limitation and the degree of dependency of the person receiving assistance, finding three levels established by the dependency law in Spain: Grade I (moderate dependency), Grade II (severe dependency) or Grade III (great dependency).^4^^,^^5^ Studies highlight the importance of distinguishing, within the clinical domain of stress, two elements: perceived stress, which refers to the extent to which various life situations are interpreted as stressful;^6^^,^^7^ and high levels of relational stress, defined by Jones *et al.*^8^ as the set of stressful life events that threaten personal relationships and generate experiences of isolation. In short, it refers to the discomfort generated by relationships with other people in the exercise of work functions.^9^Furthermore, authors such as Ajzen^10^ and Christian, Beaujean & Wright^11^ use terms like affect or affective states to refer to a concept that encompasses both emotions (defined as a state or quality of feeling at a given moment) and moods (defined as a predominant emotional tone or general attitude). Positive affect refers to positive emotional states, including motivation, creativity, and confidence. In contrast, negative affect refers to negative emotional states associated with discomfort, fear, or insecurity.^12^ Embracing this idea and conceptualizing affective states as a global concept, it is important to analyze the influence of affect on the degree of stress experienced by professional caregivers.

Psychological difficulties linked to stress, such as psychosomatic, depressive, and anxious symptoms, exhibit high comorbidity.^13^^,^^14^ Following Clark and Watson's^13^ tripartite model as an important framework, studies primarily suggest comorbidity in adults related to anxiety and depression,^12^^,^^14^highlighting the central role of affect in psychological disturbance under circumstances of high stress (considering the existence of a common substrate for anxiety and depression, which is negative affective state, explaining the extensive overlap and high comorbidity between depression and anxiety; a positive affective state (such as happiness), which in low levels characterizes depression; and a physiological state of hyperactivation (a fundamental component of fear), which characterizes anxiety).^15^ Clinical research primarily focuses on identifying variables that, when enhanced in the caregiving context, may mediate psychological difficulties, such as high levels of stress. Among these mediating variables, empathy is defined as the ability to understand, interpret, and respond to others' emotions.^16^^,^^17^ Social support refers to the set of expressive or instrumental services (which can be perceived or received) provided by trusted people, by the social networks that the person has or by the community, and which can occur both in everyday situations and in crisis situations.^18^

The literature highlights the protective role of empathy against work-related disorders such as burnout. ^19^ Studies also suggest that the use of adaptive coping styles by professional caregivers, such as problem-solving, high motivation, or emotional management, are associated with lower levels of perceived stress and greater well-being.^20^ Conversely, conditions such as lack of social support, high demands, and low control have been linked to elevated levels of stress and burnout among caregivers.^21^ Research has demonstrated that having good social support reduces the negative consequences and stress levels associated with caregiving,^22^ and positive affectivity promotes personal development in various life domains, including work and social aspects.^23^ Therefore, evidence highlights the mediating role of variables such as support and empathy in caregiving for others, particularly in the field of functional diversity. In this regard, the Person-Centered Care Model proposes a more humanistic and individualized approach to care, where a relationship of equals is built between caregiver and user, with shared decision-making and a focus on autonomy and personal development.^24^^,^^25^ Literature on this model demonstrates that promoting autonomy and positive affectivity regarding care is associated with lower levels of stress and tension in caregivers, with social support received from colleagues and during multidisciplinary work playing an important role.^26^^-^^28^

## Methods

Objectives and hypotheses. Building upon these existing concepts in the scientific literature, the objective of this study was to analyze how stress levels in professionals working in the care of people with functional diversity are influenced by the experience of positive and negative affect in these caregivers, and how this relationship is mediated by the level of empathy and social support they exhibit. The hypothesis of this study were three: (1) Positive affect will positively correlate with positive variables such as empathy and social support. Conversely, negative affect will positively correlate with higher levels of perceived stress and relational stress; (2) Positive affect will reduce the occurrence of high levels of perceived stress and relational stress, while negative affect will increase the levels of both types of stress; and (3) Social support and empathy will mediate the relationship between affect and stress, reducing levels of perceived stress and relational stress.

Study design and procedure. This was a multicentre, cross-sectional, descriptive study. The study focused on social and health care centres for people with functional diversity. The information collection process was collected between 7 June 2021 and 15 July 2021. The sample consisted of professionals in the social and health care field belonging to care centres for people with intellectual disabilities in the province of Valencia, Spain. With regard to the inclusion criteria, we looked for people who were working (between 18 and 65 years of age), and who had been working for at least one year in the social and health care field in care centres for functional diversity in the Valencian Community. With regard to the exclusion criteria, people who showed any type of mental disorder previously diagnosed at the time of data collection were excluded. 

Participants. In total, there were 756 people, of whom 78.6% (*n*=576) were women, and 21.4% were men (SD= 0.41), aged between 18 and 68 years (M=43.40, SD=11.09). Of the group of participants, 33.5% were carers (*n*=221), 27.7% were monitors (*n*=186), 1.5% were social workers (*n*=10), 10.2% were psychologists (*n*=67), 3. 6% were speech therapists (*n*=24), 9.4% were physiotherapists (n=62), 2% were social integrators (n=13), 2.7% were occupational therapists (*n*=18), and 9.4% were auxiliary services (*n*=62). The participants belonged to different centres (SD= 1.53): 8.86% belonged to early intervention centres (*n*=67); 15.08% belonged to day care centres (*n*= 114); 34.4% belonged to occupational centres (*n*= 260); 27.78% belonged to residential care centres (*n*= 210); and 5.95% belonged to sheltered housing (*n*= 45). 

Study variables. Information was collected on the demographic variables of the participating professionals, including: gender, age, professional role, job position, type of work centre, training in communication skills with people with functional diversity, and training in counselling. On the other hand, the study variables analysed were perceived stress and relational stress, psychological well-being, empathy and social support. 

Instruments. (i) To assess perceived stress, the *Perceived Stress Scale* (PSS)^5^ was used. This instrument evaluates situations perceived as stressful in daily life. It consists of 14 items with a Likert-type response format ranging from "never" (0) to "always" (4), asking participants to rate the frequency of stressful events in the past month. Scores range from 0 to 56, with higher scores indicating higher levels of stress. In this study, the questionnaire showed a reliability of α=0.73, along with adequate validity and sensitivity; (ii) To assess relational stress, the *Nurse Stress Scale*^29^was employed. This scale aims to identify the extent to which certain aspects are perceived as stressful for care or nursing staff.^30^ The questionnaire consists of 34 items with a Likert-type response format ranging from "never or almost never" (0) to "always or almost always" (4), describing various potential physical and psychological stressors. This scale demonstrated a reliability of α=0.75 in this study; (iii) Empathy was assessed using the *Empathy Quotient* (EQ^)(^^31^ in its short form validated by Redondo & Herrero-Fernández.^32^ This instrument consists of 23 items evaluating the affective and cognitive components of empathy, with a Likert-type response format ranging from "strongly disagree" (0) to "strongly agree" (3). For this research, the Cognitive Empathy dimension was studied, showing a reliability of α=0.55; (iv)To assess social support, the *Social Support Questionnaire,*^33^ adapted for the Spanish population^34^ was used. This scale evaluates the affective (expressions of love or group belonging) and confidential (possibility of having people to share concerns and problems with) dimensions of social support. It consists of 11 items with a Likert-type response format ranging from "Much less than I want" (1) to "As much as I want" (5). This questionnaire showed a reliability of α=0.85 in this study, with α=0.79 for confidential support and α=0.70 for affective support; (v) Psychological well-being, based on the affective states experienced, was assessed using the *Scale of Positive and Negative Experience* (SPANE).^35^ This instrument consists of two subscales, each with 6 items, and a Likert-type response format ranging from "rarely/never" (1) to "very often/always" (5), referring to positive affects (SPANE-P) and negative affects (SPANE-N). The reliability for SPANE-P was α=0.89 and for SPANE-N was α=0.81.

Data collection. Data collection was carried out by members of the research team. The team psychologist communicated the characteristics of the study to the participants, ensuring the commitment to confidentiality and informed consent. The application of tests and questionnaires was applied by members of the research team to those professionals who, voluntarily, wanted to participate in the study. 

Data analysis. Statistical analyses in this research utilized the SPSS 28.0 statistical package for Windows along with the PROCESS macro extension for SPSS. The following tests were conducted: Descriptive statistics (frequencies (Fr), percentages (%), means (*M*), and standard deviations (*SD*); Reliability tests (Cronbach's α) to assess the reliability of the questionnaires used; Comparison of means (Student's t-test for independent samples and One-Way ANOVA) to test hypotheses of equal means; Pearson correlation coefficient to assess the degree of relationship between variables; and PROCESS to analyze the moderating and mediating effects of two variables on the relationship between other two variables.

Ethical considerations. Regarding the ethical implications of the research, the research group undertook to sign the confidentiality commitment and inform the participants in detail, following the regulations of the 1964 Declaration of Helsinki, of the objectives of the project and what their participation will be, as well as the benefits that the results will entail. Likewise, the research group undertook to establish the requirements and conditions indicated in the Organic Law 3/2018, of 5 December, on Personal Data Protection and guarantee of digital rights, and Regulation (EU) 2016/679, of the Parliament and of the Council, of 27 April, on the protection of natural persons with regard to the processing of personal data and on the free movement of such data for the fully confidential processing of their personal data, as well as the European regulations in force. To this end, individuals will be informed, informed consent and a commitment to confidentiality will be requested.

The UV Ethics Committee has given its approval with the code UV-INV_ETICA-24773412.5**.**

## Results

### Descriptive statistics

The results obtained at the statistical level, based on mean differences, indicate significant variations primarily in empathy, perceived social support, and both perceived and relational stress. However, no significant differences were found in positive and negative affect. Differences emerged between certain groups of professionals (Table 1). For instance, in terms of empathy, caregivers and assistants exhibited the lowest levels compared to monitors, social workers, and psychologists.

Regarding social support, mean scores were highest among psychologists, with speech therapists showing particularly high levels compared to caregivers, service assistants, and monitors. In terms of stress, caregivers and monitors reported higher levels of perceived stress compared to psychologists. For relational stress, psychologists and monitors exhibited higher levels than caregivers and assistants, though lower than occupational therapists.

Considering overall scores, we examined ranges rather than absolute cut-off points. Perceived relational stress was generally moderate across all professional groups, while perceived social support was consistently high. In the case of empathy, greater variability was observed, with both high and low levels present across different professions. Finally, in terms of emotional balance, a relative equilibrium between positive and negative affect was found, with positive affect generally prevailing.

### Correlations and descriptive statistics of study variables

The correlation between cognitive empathy, social support, and the psychological variables of the study was examined. It was found that empathy significantly positively correlated with positive affect and social support, and negatively correlated with negative affect, perceived stress, and relational stress. Additionally, social support significantly positively correlated with positive affect and empathy, and negatively correlated with perceived stress and negative affect. Regarding the psychological variables, perceived stress significantly positively correlated with negative affect and relational stress, and negatively correlated with positive affect. Relational stress significantly positively correlated with negative affect and negatively correlated with empathy. The results are presented in Table 2.

**Table 1:** Descriptive statistics of the study variables according to professional role and comparative means

		C	I	SW	Psyc	ST	Phys	SI	OT	AS	F (p)	η²
Empathy	Mean	25.55	26.86	27.70	27.41	26.91	15.02	14.61	15.33	14.10	5.18 (0.001)	0.06
	SD	3.27	2.80	1.42	1.85	3.16	3.24	3.18	2.42	3.27		
	Range	14.00	16.00	4.00	8.00	10.00	12.00	8.00	7.00	11.00		
Social support	Mean	43.82	45.73	48.00	56.98	49.38	45.76	45.92	40.41	41.93	4.39 (0.001)	0.06
	SD	7.73	6.23	8.46	6.61	4.30	7.46	9.50	12.02	10.42		
	Range	42.00	30.00	22.00	21.00	15.00	34.00	24.00	30.00	32.00		
Perceived stress	Mean	23.10	22.98	21.50	19.61	22.05	23.13	23.63	24.60	22.04	2.43 (0.014)	0.03
	SD	6.64	6.10	5.25	5.51	5.80	6.51	4.48	3.87	6.04		
	Range	32.00	26.00	14.00	19.00	20.00	27.00	16.00	16.00	26.00		
Relational stress	Mean	35.84	32.95	33.75	33.73	33.71	33.51	32.60	39.14	29.50	7.02 (0.001)	0.10
	SD	7.34	5.40	6.25	5.07	5.67	5.61	6.24	4.77	4.90		
	Range	33.00	23.00	18.00	22.00	9.00	25.00	21.00	16.00	23.00		
Positive affects	Mean	18.51	18.67	19.60	19.20	19.52	18.79	20.00	18.59	18.17	1.22 (0.28)	0.02
	SD	3.30	2.47	1.96	2.24	2.09	3.05	2.86	3.02	3.43		
	Range	15.00	14.00	5.00	8.00	10.00	12.00	10.00	12.00	14.00		
Negative affects	Mean	14.90	14.66	14.00	14.00	13.63	15.02	14.62	15.33	14.11	1.07 (0.38)	0.01
	SD	3.73	3.43	2.83	2.60	4.39	2.72	2.55	3.53	3.46		
	Range	23.00	21.00	8.00	11.00	15.00	13.00	7.00	12.00	15.00		

C= Caregiver; I= Instructor; SW= Social Worker; Psyc= Psychologist; ST= Speech Therapist; Phys= Physiotherapist; SI= Social Integrator; OT= Occupational Therapist; AS= Auxiliary Services.

**Post-hoc test. *Emphaty*:** Caregiver < Instructor, Social Worker and Psychologist; Instructor, Social Worker and Psychologist > Auxiliar Services. ***Social support*:** Caregiver < Psychologist and Speech Therapy; Instructor < Speech Therapy; Speech Therapy > Auxiliar Services. ***Perceived stress*:** Caregiver, Instructor > Psychologist. ***Relational stress*:** Caregiver < Instructor and Auxiliar Services; Instructor and Psychologist > Auxiliar Services; Instructor and Psychologist < Occupational Therapist. ***Positive affects*:** no effects. ***Negative affects*:** no effects.

**Table 2:** Correlations and descriptive statistics of study variables

	Empathy	Social support	Perceived stress	Relational stress	Positive affects	Negative affects
Empathy	1					
Social support	0.207**	1				
Perceived stress	-0.285**	-0.378**	1			
Relational stress	-.0119**	-0.079	0.267**	1		
Positive affects	0.184**	0.417**	-0.471**	0.001	1	
Negative affects	-0.177**	-0.267**	0.581**	0.291**	-0.410**	1
*Mean*	26.41	45.24	22.71	33.88	18.81	14.69
*Standard Deviation*	2.82	7.33	6.38	6.19	2.83	3.40
*Range*	16.00	42.00	32.00	34.00	15.00	24.00
*Skewness (A)*	-0.580	-1.21	0.249	0.540	-0.367	0.692
*Kurtosis (K)*	0.127	1.733	-0.248	0.361	0.393	1.78

*p≤0.05; **p≤0.01; ***p≤0.001

### Mediation models between affective states and stress, with empathy and social support as mediating variables

Four mediation models were conducted to determine if cognitive empathy and social support mediated the relationship between positive and negative affects, and perceived and relational stress. Variables with the highest correlation were selected for these models.

**Figure 1:**
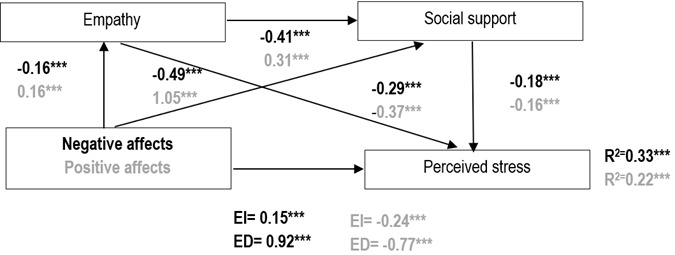
Mediation model for perceived stress

*Affects and perceived stress.* In the mediation study between negative affects and perceived stress, negative affect negatively influenced empathy (*β* = -0.16) and social support (*β* = -0.49). In turn, empathy negatively affected social support (*β* = -0.41) and perceived stress (*β* = -0.29), and social support negatively affected perceived stress (*β* = -0.18). Additionally, the direct effect of negative affects on perceived stress was positive (*β* = 0.92), while the indirect effect through empathy and social support was positive but significantly lower (*β* = 0.15). This mediation explained 33% of the variance in perceived stress. For positive affects and perceived stress, positive affect positively influenced empathy (*β* = 0.16) and social support (*β* = 1.05). Empathy positively affected social support (*β* = 0.31) and negatively affected perceived stress (β = -0.37), while social support negatively influenced perceived stress (*β* = -0.16). The direct effect of positive affects on perceived stress was negative (*β* = -0.77), with the indirect effect through empathy and social support also negative but significantly lower (*β*= -0.24). This mediation explained 22% of the variance in perceived stress. The results are presented in Figure 1.

*p≤0.05; **p≤0.01; ***p≤0.00; Black color: values related to negative affect; Gray color: values related to positive affect

**Figure 2:**
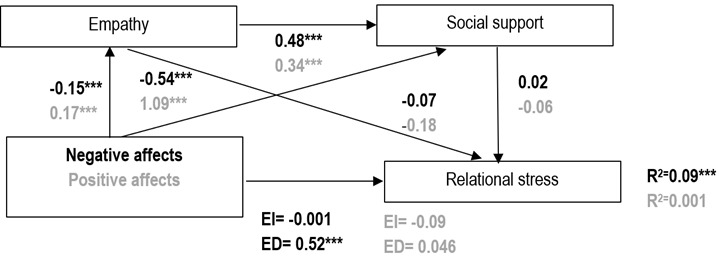
Mediation model for relational stress

*Affects and relational stress.* In the mediation study between negative affects and relational stress, negative affect negatively influenced empathy (*β* = -0.15) and social support (*β* = -0.54). Empathy positively affected social support (*β*=0.48) and negatively affected relational stress (*β* = -0.07), while social support had a non-significant positive effect on relational stress (*β* = 0.02). The direct effect of negative affects on relational stress was positive (*β* = 0.52), while the indirect effect through empathy and social support was negative and non-significant (*β* = -0.008). This mediation explained 9% of the variance in perceived stress. For positive affects and relational stress, positive affect positively influenced empathy (*β=0*.17) and social support (*β* = 1.09). Empathy positively affected social support (*β* = 0.34) and negatively influenced relational stress (*β* = -0.18), while social support negatively influenced relational stress (*β* = -0.06). The direct effect of positive affects on relational stress was positive (*β* = 0.046), with the indirect effect through empathy and social support negative but non-significant (*β* = -0.09). This mediation explained 0.06% of the variance in perceived stress. The results of these medications are shown in Figure 2.

*p≤0.05; **p≤0.01; ***p≤0.001; Black color: values related to negative affect; Gray color: values related to positive affect

## Discussion

The performance of professional tasks in caring for individuals with functional diversity entails a series of implications that can exacerbate psychological clinical issues related to high levels of stress. This experience of stress will be influenced by mediating variables,^18^ making it crucial to detect and address them adaptively in caregiving contexts. For this reason, the aim of this research was to analyze how the affective states of professionals working as caregivers for individuals with functional diversity are related to the perceived stress and relational stress they experience, and how empathy and social support may mediate this relationship. Firstly, *hypothesis 1* suggested that positive affect would positively correlate with empathy and social support, while negative affect would positively correlate with perceived stress and relational stress. The results of this research are consistent with this hypothesis since positive affect significantly and positively correlated with social support and empathy in its cognitive dimension, and negatively with perceived stress, relational stress, and negative affect. Conversely, negative affect significantly and positively correlated with perceived stress and relational stress, and significantly negatively with positive affect, empathy, and social support.

These findings are in line with the scientific literature, which emphasizes the role of positive affect as a protective factor against high levels of perceived stress,^18^ and the importance of a life linked to reliable sources of social support, an empathetic attitude, and low negative affect in reducing stress and the negative consequences of caregiving, as well as in promoting healthy personal development.^3^^,^^4^^,^^17^^,^^20^ Secondly, *hypothesis 2* suggested that positive affect would be negatively related to elevated levels of perceived and relational stress, while negative affect would be positively associated with higher levels of perceived and relational stress. The results of the present research partially support this hypothesis, as positive affect had a significantly negative effect on perceived stress but had little effect on relational stress, which was not significant; meanwhile, negative affect had a significantly positive effect on perceived stress and relational stress. These data are consistent with those in the literature, explaining that negative affect and its characteristics are related to high levels of stress and burnout in professional caregivers,^27^ while positive affect promotes better professional and social development and lower levels of stress,^21^ as well as a greater interest and quality of care provided by professionals assisting individuals with functional diversity.^3^

Finally, *hypothesis 3* proposed that social support and empathy would mediate the relationship between affect and stress, reducing levels of perceived and relational stress. The results of our research partially supported this hypothesis. Firstly, it was observed that in the case of negative affects, cognitive empathy, and social support mediated the relationship with perceived stress, significantly reducing its levels. Regarding positive affects, empathy, and social support also significantly reduced the levels of perceived stress, although to a lesser extent than in the case of negative affects. In contrast, it was observed that, although for both negative and positive affects, empathy and social support reduced levels of relational stress, this reduction was not significant, so they did not influence the reduction of relational stress levels and did not act as mediating variables between this and affective states. In this regard, the results were consistent with the existing evidence in the literature, which explains that lack of social support is related to high levels of stress,^19^ while having good social support and being an empathetic person act as protective factors against high levels of stress.^17^^,^^24^^-^^26^ Additionally, these variables acted as protective factors in professionals who assist as caregivers for individuals with functional diversity, promoting positive self-esteem and greater satisfaction with their work.^3^

Although this study highlights the importance of mediating variables in regulating stress levels among professionals caring for individuals with functional diversity, it has some limitations. Firstly, it's a cross-sectional study, conducted at a single point in time, suggesting the need for future longitudinal research to explore these findings over a longer period. Additionally, participants were solely from centers in the Valencian Community in Spain, limiting generalizability. Therefore, expanding the sample to include different roles and replicating the study across various regions and countries would strengthen the conclusions. It should also be noted that the sample is not highly representative, so it would be important to increase the size and heterogeneity of the sample in order to obtain better results. In addition, the reliability of the instruments used is medium-low, so it would be advisable to use tests with higher reliability. Furthermore, the study only assessed empathy from its cognitive aspect, suggesting further exploration of its emotional dimension. Finally, there's a lack of distinction in the literature between perceived stress and relational stress, highlighting the need to clarify these variables.

While acknowledging the outlined limitations, this study underscores the significance of fostering empathy and cultivating robust social support networks among professional caregivers. These variables, in their mediating capacity, mitigate the diverse stressful situations and associated emotional distress encountered by these professionals. For this reason, it is important to promote strategies and actions in social care settings aimed at reducing the stress levels associated with caregiving, in addition to promoting personal adjustment in professionals and encouraging the presence of these protective mediating variables. In this sense, efforts should be made to establish a quality relationship between these professionals and users, promoting shared decision-making and a more humanized care approach, and nurturing personal growth and autonomy both in users and in the professional caregivers who assist them.

Conclusion. The aim of the present research was to analyse how the stress levels of professionals working in care for people with functional diversity are influenced by positive and negative affect, and how this relationship is mediated by the level of empathy and social support they present. Thus, the results of this research showed that positive affect correlated significantly positively with social support and empathy, and correlated negatively with high levels of perceived and relational stress. Conversely, negative affect correlated significantly positively with elevated levels of perceived stress and relational stress, while it correlated negatively with empathy and social support. 

Furthermore, positive affect significantly influences the reduction of high levels of perceived stress, while its effect on relational stress is practically null. On the other hand, negative affect significantly influences the increase of perceived stress levels, and significantly influences the increase of relational stress levels, although in this case, the influence is smaller. Finally, it was observed that empathy and social support significantly mediated the relationship between affect and perceived stress, decreasing the levels of perceived stress, while the mediating effect of these variables between affect and relational stress was not significant, and therefore had little influence on the relationship between the two variables. 

From a practical perspective, these findings highlight the need to implement continuous training programs for healthcare professionals that strengthen empathy and foster the development of social support networks. Enhancing these aspects can be crucial in reducing both perceived and relational stress, ultimately improving well-being and the quality of patient care. It is essential to consider the specific roles of different healthcare professionals, as their functions and responsibilities may shape their experiences of stress and social support. Although this study did not focus exclusively on nursing, the findings are highly relevant to this group, given the similarities in roles between nurses, nursing assistants, and other direct care professionals. Like their colleagues, nurses are exposed to emotionally demanding situations and interpersonal stressors, making them equally susceptible to the effects observed in this study. Therefore, promoting empathy, strengthening peer support networks, and addressing profession-specific challenges should be key priorities in healthcare training and organizational policies.

## Declaration of data availability

The data that support the findings of this study are available from the corresponding author, [SVM], upon reasonable request.
